# Electrically-Evoked Proximity Sensation Can Enhance Fine Finger Control in Telerobotic Pinch

**DOI:** 10.1038/s41598-019-56985-9

**Published:** 2020-01-13

**Authors:** Ziqi Zhao, Minku Yeo, Stefan Manoharan, Seok Chang Ryu, Hangue Park

**Affiliations:** 1 Department of Electrical & Computer Engineering, Texas A&M University, College Station, TX 77843 USA; 2 Division of Mechanical and Biomedical Engineering, Ewha Womans University, Seoul, 03760 South Korea

**Keywords:** Sensory processing, Electrical and electronic engineering

## Abstract

For teleoperation tasks requiring high control accuracy, it is essential to provide teleoperators with information on the interaction between the end effector and the remote environment. Real-time imaging devices have been widely adopted, but it delivers limited information, especially when the end effectors approach the target following the line-of-sight. In such situations, teleoperators rely on the perspective at the screen and can apply high force unintentionally at the initial contact. This research proposes to deliver the distance information at teleoperation to the fingertips of teleoperators, *i.e*., proximity sensation. Transcutaneous electrical stimulation was applied onto the fingertips of teleoperators, with the pulsing frequency inversely proportional to the distance. The efficacy of the proximity sensation was evaluated by the initial contact force during telerobotic pinch in three sensory conditions: vision only, vision + visual assistance (distance on the screen), and vision + proximity sensation. The experiments were repeated at two viewing angles: 30–60° and line-of-sight, for eleven healthy human subjects. For both cases, the initial contact force could be significantly reduced by either visual assistance (20–30%) or the proximity sensation (60–70%), without additional processing time. The proximity sensation is two-to-three times more effective than visual assistance regarding the amount of force reduction.

## Introduction

In dexterous telerobotic operations, the structure of robotic end effectors has evolved from a simple rod into a complex gripper, which resembles human fingers to provide enhanced intuitiveness and control accuracy. The grippers generally follow the motion input from the handle grasped by human fingers at the master interface, as introduced in the typical master-slave surgical robotic systems^1–3^. The interface has been designed to provide a kinematic transparency between the operator’s fingers and the robotic gripper tips, so that the teleoperator can feel the gripper tips as one’s own, extended fingers.

**Figure 1:**
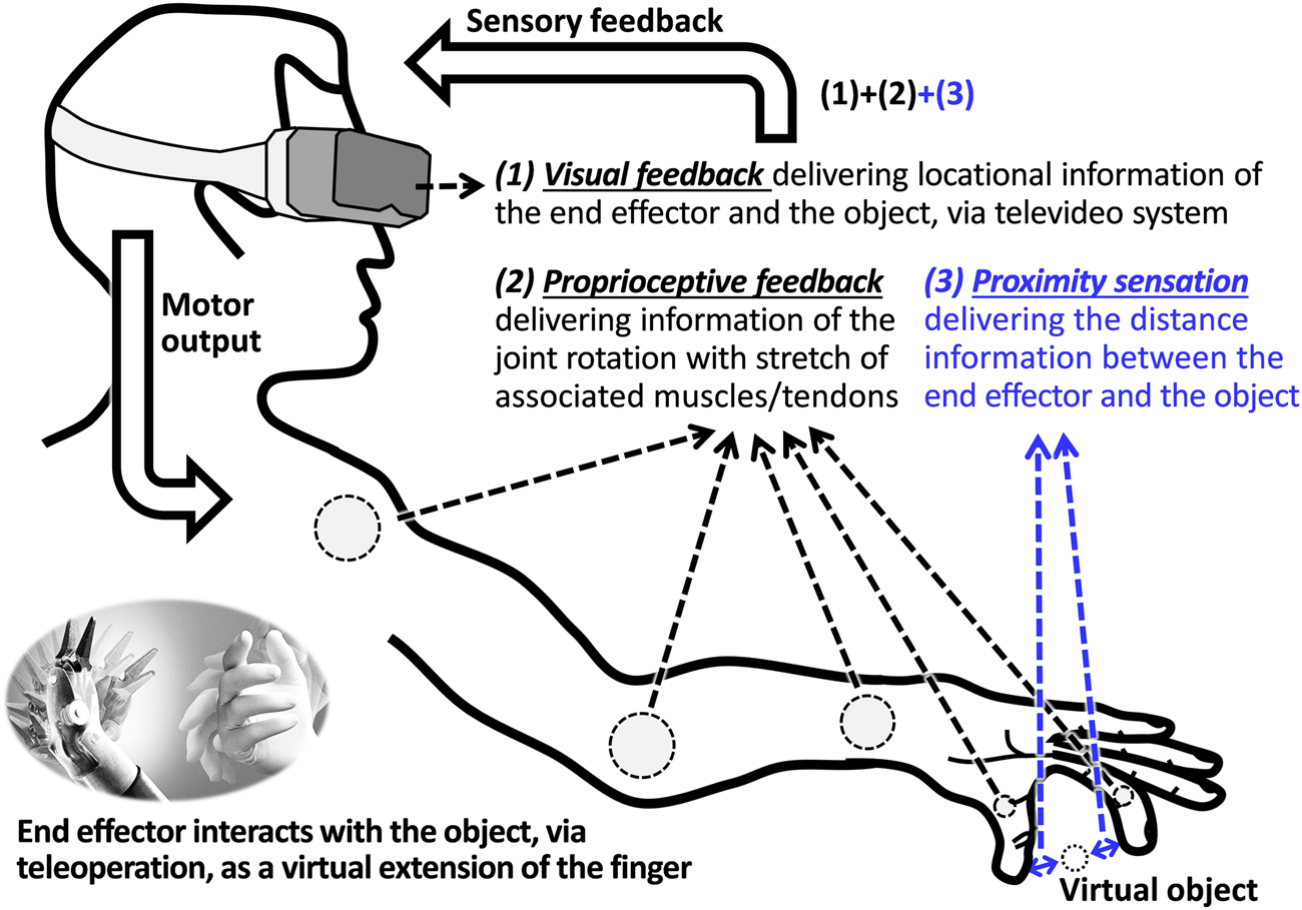
Overall block diagram of sensorimotor loop for the telerobotic pinching operation, with the representation of associated sensory feedback: (1) visual feedback, (2) proprioceptive feedback, and (3) proximity sensation proposed in this paper.

Human finger motion for physically interactive tasks can be categorized into three phases: approach, contact, and interact in the order of time. For each phase, a different set of peripheral sensory feedback is provided to the human nervous system and helps human operators secure a certain level of control accuracy. Although visual feedback plays a major role in delivering sensory information during human motor control with its incomparable information transfer capability, peripheral sensory feedback effectively compensates for limitations of visual feedback. For example, tactile feedback from the fingertips provides texture and pressure during the contact and interact phases, which can be only roughly detected by visual feedback. Proprioception at the muscles proximal to the fingertip is commonly employed throughout all the phases and provides useful spatial information, especially during the approach phase as the fingertip delivers very limited information before contact occurs, such as temperature or air flow^4^. These roles of peripheral sensory feedback can be even more important for telerobotic operations as shown in Fig. 1, because of the limited visual feedback delivered through the televideo system.

The proprioception, however, is not reliable for the fine fingertip control because of the considerable distance between the joints along the arm and fingertips^5,6^. A small estimation error in using the proprioceptive feedback or the intrinsic kinematic parameters of the upper-limb saved in the brain can cause severe error at the fingertip. Therefore, a teleoperator usually relies heavily on visual feedback, especially during the approach phase, leading to the integration of advanced vision algorithms with high-definition sensors. Despite that, visual feedback cannot be an ultimate solution because it delivers a limited amount of information when the gripper tips and target are aligned in the line of sight. Also, in surgical teleoperation, an endoscopic camera is always located behind two or more grippers^7,8^ to form triangulation. When the vision is blocked or unclear due to anatomical structures, blood, or instruments, which is often the case, the camera cannot capture the approaching motion properly. Further, understanding visual feedback for use in fingertip manipulations needs significant cognitive involvement and processing time to interpret the real-time video, which can be a high burden to the teleoperators who need to handle multiple tasks demanding continuous decision making during the teleoperation. The intrinsic visual-proprioceptive mapping error is another challenge, because even the high-resolution visual feedback may not be effective with the visual-proprioceptive mapping error^9,10^.

The lack of reliable sensory feedback during the approach phase can result in higher force than the desired at the moment of contact between the end effector under teleoperation and the target objects, potentially disturbing subsequent manipulations or permanently damaging sensitive objects^11,12^. A high level of concentration is required to address this issue^13,14^, but it is still challenging to accurately estimate the necessary information and to complete the task on a timely basis. As an alternative, visual assistance displaying texts or numbers on the screen and virtual reality have shown their efficacy for fine force control during robotic surgery^15–19^. The efficacy of visual assistance in improving control accuracy can be further enhanced by intuitive display (*e.g*., graphical display). Graphics, however, still occupy visual feedback, which can negatively affect the teleoperator’s concentration on the target site. A similar problem exists when using auditory feedback as an assistance, as auditory feedback is often occupied by verbal communications. Also, its efficacy as an assistance is limited by the coding capacity of the human auditory system and its nature optimized to process either natural sounds or speech^20,21^.

Peripheral sensory augmentation usually requires less cognitive load than visual or auditory does to deliver sensory information^21,22^. Thus, it could be another way to compensate for limited sensory feedback during the approach phase of the teleoperation, which has high accuracy needs. Also, it is not as heavily occupied in daily life as visual or auditory feedback. Haptic feedback is one of the most promising candidates for such peripheral sensory augmentation in telerobotic applications^23–25^ because it uses the immense potential of the periphery for the reception of sensory information. However, so far in most cases, haptics has not been provided during the approach but instead during the contact and interact phases. For example, haptic interface devices were developed to let the user feel the stiffness of the tissue at dissection, via the information from a multi-axis force and torque sensor attached to the cutting instrument^26^. Most commercial haptic interfaces showed their efficacy in distinguishing between different tissues during the contact and interact phases^27–29^. Although a few examples of haptic feedback have been integrated during the approach phase^16,17,30,31^, all of them were designed to deliver the predefined boundaries of a safe workspace^32–34^, rather than providing real-time sensory information during the approach.

In this paper, we propose to provide a proximity sensation directly on the fingertips during the approach phase, by employing an electrically-evoked pulsing sensation that can be implemented in a small form factor, suitable for human fingertips. The proposed proximity sensation will allow teleoperators to enjoy the advantage of high resolution and rich cortical engagement of the human fingertip, which is one of the body parts with the highest level of sensory resolution as demonstrated in several studies and represented by the large corresponding somatosensory cortex area^35–37^. The effectiveness of the sensation was evaluated experimentally for a telerobotic pinching operation, where the distance between the robotic gripper tip and a target object was fed back to the operator in real-time, *i.e*., proximity sensation, as a continuous electrical pulse at the frequency inversely proportional to the distance.

## Goal and Hypotheses

The overall goal of the current research was to determine the effect of the proposed fingertip proximity sensation on telerobotic grasping and manipulation of a remote object with finger-like slave grippers, especially when the visual feedback provides limited information. *The main hypothesis* was that the proximity sensation between the fingertip and the target object would help the operator to reduce the initial contact force of telerobotic pinching if necessary. Since a lower contact force initiates the contact phase in a more stable way, task performance potentially can be improved via a safer and smoother transition toward subsequent manipulation. We expected that the effect of the proximity sensation would be more prominent when the object was in the line of sight, because limited visual information makes it hard to control the initial contact force. *The second hypothesis* was that the proximity sensation would be more effective than visual assistance provided by numbers, on minimizing the initial contact force in telerobotic pinching. The proximity sensation is direct and intuitive, as it is applied to the fingertip corresponding to the end effector that is interacting with the target object, while visual assistance needs an additional visual-proprioceptive mapping between the numbers on the screen (*i.e*., distance) and the proprioceptive feedback in charge of following the fingertip movement. Also, visual assistance needs to share visual resource that is heavily occupied by the observation of the end effector.

## Implementation of the Proximity Sensation

### Implementation of the proximity and tactile sensations together on the fingertip

To evoke proximity sensation on the fingertip, transcutaneous electrical stimulation onto the palmar digital nerve was selected with a stimulation frequency inversely proportional to the distance between the robotic tool and the targeted object. Electrical stimulation was selected due to its smaller form factor compared to that of mechanical stimuli, which need several moving parts to interact with the fingertip^38,39^. The inversely-proportional relationship between the frequency and the distance was selected to follow the practice of existing applications that use frequency-modulated sensory interventions in delivering distance information^40,41^. The location of stimulation, the distal part of the palmar digital nerve, is composed mostly of cutaneous axons and located at the bony area with minimal fats and muscles near to the skin. Therefore, transcutaneous electrical stimulation of the skin over this nerve easily elicits the electrotactile feedback on the fingertip^42,43^. Since each subject has a different perceptual sensitivity to the stimulation frequency, the perceptible range of each subject was tested and used to find the optimal frequency range. First, the minimum frequency was set to 10 *Hz* for the information transfer rate to be reasonable (*i.e*., deliver pulsing sensation for every 100 *ms*), while maximizing the usable frequency range. Second, the maximum frequency for the proximity sensation was set to 5 *Hz* lower than the highest frequency (*f*_*max*_) that evokes pulsing sensation. A 5 *Hz* margin was added to make sure that subjects felt the pulsing sensation over the entire frequency range used to evoke proximity sensation.

**Figure 2:**
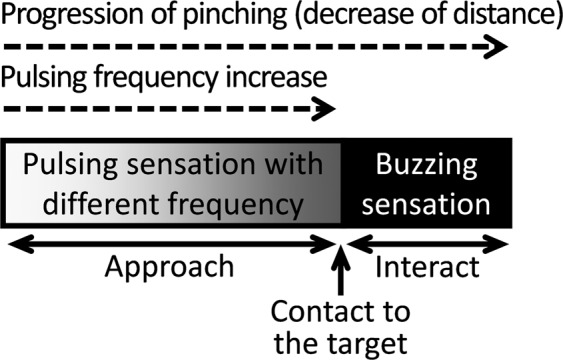
Sensory feedback on the teleoperator’s fingertip during the entire phase of the telerobotic pinching operation (*i.e*., approach, contact, and interact), according to the progression of pinching (decrease of distance).

It is also important to integrate the tactile sensation together with the proximity sensation on the fingertip, considering that the pinching operation evokes tactile feedback after the contact is made. By integrating both the proximity and tactile sensations, the sensory feedback on the fingertip can be continuous over the entire phase of the pinching operation. When the fine motor control at the fingertip is crucial at contact, the continuity of sensory feedback at contact would be important to minimize the unwanted overshoot or undershoot of motor output at contact. To deliver both proximity and tactile sensations during a telerobotic pinch, the electrotactile feedback should be changed discretely at contact, for the nervous system not to become confused^44^ between the two sensations. When the stimulating frequency is above certain threshold, our nervous system can no longer feel the pulsing and will instead feel buzzing. As this buzzing-type electrotactile feedback is clearly distinguishable from the pulsing-type electrotactile feedback, we selected pulsing and buzzing sensations, respectively for proximity and tactile sensations, as shown in Fig. 2. To evoke buzzing sensation on the fingertip after the contact was made at teleoperation, we programmed the stimulation frequency to be increased discretely by 10 *Hz*, from *f*_*max*_ − 5 *Hz* to *f*_*max*_ + 5 *Hz*, when the contact is made. With this discrete change of frequency and the following discrete change of electrotactile feedback, we could provide sensory discrepancy between the approach and contact phases, with pulsing during the approach phase and buzzing during the contact phase. During the interact phase after contact, the stimulation frequency stayed the same, so as not to deliver further information and purely evaluate the efficacy of the proximity sensation at the initial contact.

### Implementation of the proximity sensation onto the telerobotic pinch via the robotic gripper

**Figure 3:**
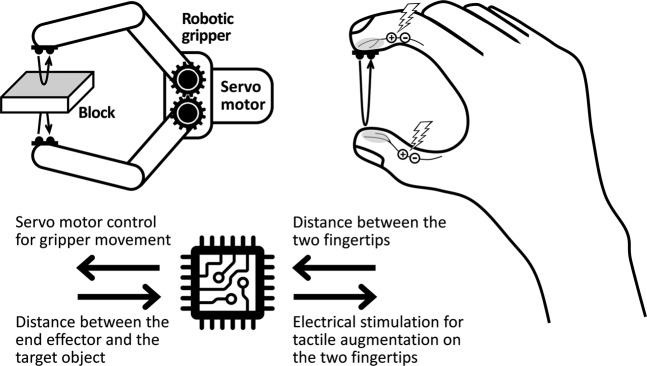
Graphical representation of the experimental setup: An optical distance sensor, installed onto the silicone fingertip guard and worn on the fingertip, will measure the distance between the thumb and the index finger. The processed data will be sent to the gripper motor to control the gripper movement. Two optical distance sensors, installed on each side of the gripper tip, will detect the distance between the gripper tip and the target object. The distance information will be delivered to teleoperator’s fingertip by transcutaneous electrical stimulation, with the pulsing frequency inversely proportional to the distance.

The telerobotic pinching operation was selected to test the effectiveness of the proximity sensation in telerobotic object manipulation because it is the most basic mode of fine finger control^45–48^ and has been widely used for telerobotic surgeries^49,50^. Also, tips of the robotic gripper move along one direction, making the data analysis and comparison simple and clear. The sensorimotor interface used in this study allowed bi-directional communication between the teleoperator’s finger and the robotic gripper. *First, as a motor pathway from the finger to the gripper* (Fig. 3), the servo motor of the gripper was controlled by the output of an optical distance sensor installed on the index finger via a silicone fingertip guard, which provided the information of distance between the thumb and the index finger. The distance information between the two fingers was subsequently transferred to the servo motor that controls the gripper tips, via a motor control signal with a pulse width modulation. As the distance between the subject’s thumb and index finger becomes smaller, the gripper tips approach each other to a corresponding distance, and vice versa. Note that the movement of the gripper tip is discrete based on the specification of the servo motor, and the resolution is 60 *µm*. *Second, as a sensory pathway from the gripper to the finger* (Fig. 3), the distance information between each of the gripper tips and the object was measured by the optical distance sensor and delivered to the fingertips of the thumb and the index finger in the form of a pulsing sensation (*i.e*., proximity sensation). The frequency of electric stimulus was inversely proportional to the distance from each of the gripper’s fingertips to the object. The relationship between the sensor value (*i.e*., distance) and the stimulation frequency (*f*_*stim*_) is described in the equation below.

$${f}_{stim}=10+\frac{{d}_{open}-{d}_{current}}{{d}_{open}-{d}_{close}}\{({f}_{max}-5)-10\}$$

where *f*_*max*_ is the maximum frequency of each subject for pulsing sensation, *d*_*open*_ and *d*_*close*_ are the optical distance sensor readings corresponding to the distance between gripper tips when the gripper is fully open and fully closed, respectively (*i.e*., *d*_*open*_ = *15* *mm*, *d*_*closed*_ = 0 mm), while *d*_*current*_ is the current distance between gripper tips and the target object. According to the equation, the subject is expected to feel a slow pulsing sensation (*f*_*stim*_ = 10 *Hz*) at the maximum distance between the gripper tips and the target object and a fast pulsing sensation (*f*_*stim*_ = (*f*_*max*_ − 5) *Hz*) right before the contact is made.

## Results

### Comparison of the initial contact force among Vision only, Vision + Visual Assistance, and Vision + Proximity Sensation, in two viewing angles

**Figure 4:**
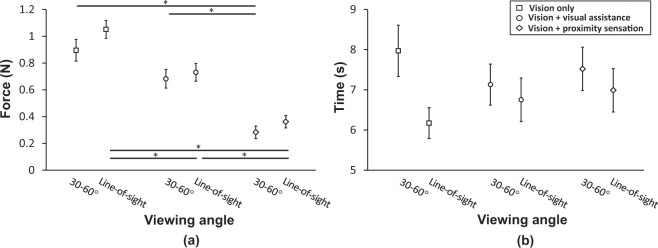
Comparison of the telerobotic pinching performance according to the condition of sensory feedback and viewing angle: (**a**) initial contact force applied onto the target object and (**b**) approach time that subjects spent to control the gripper to touch the target object. Error bar indicates the standard error and asterisk (*) indicates statistical difference with 95% confidence interval via two-tailed Welch’s t-test with Bonferroni correction.

As seen in Fig. 4a, with the proximity sensation, subjects could lower the initial contact force by 68.4% at 30–60° viewing angle and 65.6% at a line-of-sight viewing angle (p < 0.001 for both viewing angles), compared to the initial contact force with only visual feedback. The significant reduction of the initial contact force with the proximity sensation supports the main hypothesis that the proximity sensation between the fingertip and the target object would help the operator to reduce the initial contact force of telerobotic pinching when necessary.

With visual assistance, subjects could lower the initial contact force by 23.8% and 30.5% at 30–60° and line-of-sight viewing angles, respectively. However, the reduction at the 30–60° viewing angle is not statistically significant (p = 0.047) while the reduction at the line-of-sight viewing angle is statistically significant (p < 0.001). For both viewing angles, the amount of force reduction with the proximity sensation was 2 × −3x times larger than the force reduction with the visual assistance, both compared to the force applied in the vision-only condition. These results support our second hypothesis that the proximity sensation would be more effective than visual assistance provided by numbers, in minimizing the initial contact force for telerobotic pinching.

### Comparison of the approach time among Vision only, Vision + Visual Assistance, and Vision + Proximity Sensation, in two viewing angles

In both the 30–60° and line-of-sight viewing angles, no statistically-meaningful time difference was observed between any two conditions of sensory feedback, as shown in Fig. 4b. In other words, the approach time did not change by the condition of sensory feedback. The approach time did not change by the viewing angle either, based on the comparison under the same sensory condition. At the vision-only condition, although it was not statistically significant, subjects tend to spend less time with the line-of-sight viewing angle than with the 30–60° viewing angle (p = 0.024).

### Amplitude/Frequency range for each subject

**Figure 5:**
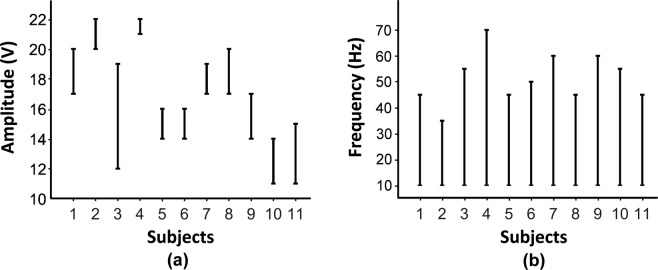
(**a**) Voltage amplitude ranges evoking comfortable electrotactile feedback and (**b**) frequency ranges evoking pulsing sensation, for each of eleven subjects.

Fig. 5a summarizes the usable voltage ranges to deliver electrotactile feedback for each of eleven subjects. Note that, we recorded the minimum voltage level when subjects reported any sensation (*i.e., perception threshold*), and recorded the maximum voltage level when subjects reported any discomfort from the stimulation (*i.e., comfort threshold*). When setting the frequency of the stimulus at 100 Hz, the average perception threshold was 15.3 *V* with a standard error (STE) of 1.03 *V* and the average comfort threshold was 18.2 *V* with an STE of 0.829 *V*. Fig. 5b summarizes the usable frequency range to deliver pulsing sensation for each of eleven subjects. When setting the amplitude of the stimulus at the mean value of the perception and comfort thresholds collected for each person, all subjects could feel a pulsing sensation from 10 *Hz* and the average maximum frequency for subjects to feel a pulsing sensation was 51.4 *Hz* with an STE of 2.95 *Hz*. To make sure the frequency range for pulsing sensation was consistent during the experiment, we confirmed that the frequency range did not change after the whole experiment for each subject.

### Results of the questionnaire to evaluate the perception of electrotactile feedback according to the stimulation frequency

**Figure 6:**
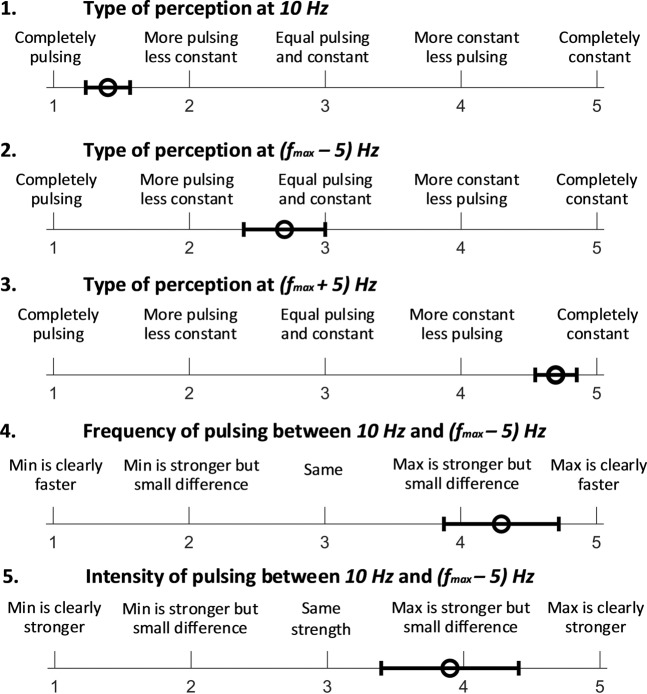
Five questions of the questionnaire to evaluate the perception of the electrotactile feedback: Question 1–2 are to evaluate the perception of the electrotactile feedback at marginal values of frequency range for pulsing sensation, Question 3 is to confirm the effectiveness of using the frequency of (f_max_ + 5) *Hz* to evoke the buzzing sensation, and Question 4–5 are to confirm the effectiveness of frequency modulation at the frequency range between 10 *Hz* and (f_max_ − 5) *Hz*. The white circles indicate the average and the error bar indicates the standard error.

Fig. 6 shows the results of the questionnaire, with the average and standard error among the data from eleven subjects. Results of question 1 suggest that subjects felt very clear pulsing sensation at 10 *Hz*. Results of question 2 suggest that, at (*f*_*max*_ − 5) *Hz*, subjects felt both pulsing and buzzing sensation, somewhat closer to pulsing. Results of question 3 suggests that subjects mostly felt a constant buzzing sensation instead of pulsing sensation at (*f*_*max*_ + 5) *Hz*. Results of question 4 suggest that subjects clearly felt the difference in pulsing frequency between the pulsing at (*f*_*max*_ − 5) *Hz* and the pulsing at 10* Hz*. Results of question 5 suggest that subjects felt a higher intensity of pulsing sensation at (*f*_*max*_ − 5) *Hz* than at 10 *Hz* but with a small difference.

## Discussion

### Tactile feedback on the fingertip can be an efficient information modality to deliver the distance information during the approach phase of telerobotic pinch

Tactile feedback on the fingertip is not normally engaged in the approach phase of telerobotic pinch, as no contact has been made between the fingertip and the object. The most important message that this paper delivers to the readers is the immense potential of tactile feedback on the fingertip to be used as a proximity sensation to improve the control accuracy of the initial contact force. The addition of proximity sensation could decrease the initial contact force by 60–70%. The comparison between the effects of proximity sensation and visual assistance also demonstrates that the tactile feedback on the fingertip (as a proximity sensation) is very “effective” to deliver the varying distance information during the approach phase of a telerobotic pinch, considering that visual feedback has been considered as the most effective modality to deliver information.

### The efficacy of visual assistance is variable among subjects, perhaps because of the different visual-proprioceptive coordination capability, while the effect of the proximity sensation is consistent over all subjects, perhaps because of the consistent electrotactile-proprioceptive coordination capability

By examining the data in more detail, we found that some of the subjects (4 of 11 at the 30–60° viewing angle and 2 of 11 at the line-of-sight viewing angle) applied even higher initial contact force on average with visual assistance, compared to the force applied to the visual-only condition. On the other hand, all subjects applied lower initial contact force with the proximity sensation, compared to the force applied with the visual-only condition. We expect that those 2–4 subjects, for whom visual assistance was not at all effective for telerobotic pinching, have poor visual-proprioceptive coordination. It is perhaps because the efficacy of visual assistance would depend heavily on the visual-proprioceptive mapping capability of each subject (*i.e., the* mapping between visual feedback and proprioceptive feedback on muscles associated with pinching)^9,10,51^. Another possible interpretation is that visual assistance could not be used effectively in real-time telerobotic pinching, because the visual feedback was heavily engaged in the observation of the robotic gripper. On the other hand, all 11 subjects could use the proximity sensation effectively to reduce the initial contact force, which suggests that electrotactile-proprioceptive mapping (*i.e*., mapping between the electrically-evoked proximity sensation on the fingertip and proprioceptive feedback on the muscles associated with pinching) is consistent over subjects.

### Neither the proximity sensation nor visual assistance increases approach time

As shown in Fig. 4b, it was observed that the average time that subjects spent to approach the object (*i.e*., approach time) did not change when adding either the proximity sensation or visual assistance. This result suggests that both the proximity sensation and visual assistance provide distance information to the teleoperator in an intuitive way that requires minimal processing time, although further study is needed to test the cognitive involvement. Despite no statistical significance, the approach time tends to somewhat decrease with either the proximity sensation or visual assistance, at the 30–60° viewing angle. We also cannot exclude the possibility that the approach time can be further reduced after the teleoperator learns how to use the additional sensory information.

### Electrically-evoked pulsing sensation on the fingertip conveys analog information intuitively to human operator, if the stimulation parameters are well customized for each subject

The results of the questionnaire indicate that subjects could feel the difference in the frequency of stimulation, via both frequency and intensity of pulsation, if the frequency was within the range for pulsing sensation (see Fig. 6). Therefore, we expect that the frequency modulation, within the frequency range for pulsing sensation, can be used to deliver analog information. It agrees with previous studies regarding the immense potential of electrotactile feedback on the fingertip in sending multi-bit digital information via frequency modulation^52–55^. Based on the questionnaire results, subjects could distinguish the pulsing sensation from the buzzing sensation and the pulsing sensation can be discretely changed to the buzzing sensation by a leap of 10 *Hz* from (*f*_*max*_ *− 5*) *Hz* to (*f*_*max*_ + *5*) *Hz*. This result suggests that pulsing and buzzing sensations can be used together in a single system to deliver different information. For example, the pulsing sensation with frequency modulation can deliver the distance at proximity and the buzzing sensation can deliver the discrete on/off state for contact.

Additionally, it was found that both the perception threshold of stimulation amplitude and the range of pulsing frequency are variable among subjects, as seen in Fig. 5. This result suggests that the stimulation parameters should be customized for each teleoperator based on the perception test before the stimulation is used to evoke electrotactile feedback. However, it is still possible to define a common frequency range for pulsing, generally applicable to all subjects^56–60^, as all subjects reported a pulsing sensation at a frequency range between 10 and 35 *Hz*.

### Inversely-proportional relationship between frequency and distance works well, but further investigation is needed to find the best modulation method to deliver the distance information

Humans feel an object is approaching when the frequency of the sensory feedback is increasing^40^. This perhaps is related to the Doppler effect that explains the frequency increase of sound, light, or other waves as the source and observer move toward each other. Such a perception has been applied to the parking assistant system of cars, which generates a warning sound that beeps with higher frequency as the exterior of the car approaches a wall^41^. Similarly, the sensory feedback with higher frequency could be mapped intuitively to the closer distance between the fingertip and the object. We found that this relationship worked well in the proposed telerobotic pinch system, considering that the proximity sensation reduced the initial contact force, as intended.

However, it has not been tested whether the inversely-proportional relationship between frequency and distance is the best option to deliver the distance information. Although the frequency or amplitude modulations have been developed to work efficiently with the electrical system, the best option for the neural communication might be different. We expect that another effort is necessary to find the neural-friendly code to deliver the distance information effectively and intuitively. Also note that we excluded the option of amplitude modulation in delivering distance information for two reasons: 1) the range of usable amplitude was very narrow for some subjects, as they felt discomfort right above their perception threshold (see Fig. 5), and 2) there is an adaptation issue when a stimulation is applied for a long time period (*i.e*., the amplitude of stimulation needs to be increased as the nervous system adapts to the stimulation).

### Further investigation is needed to show the viability of this approach if the object is not located exactly in between two gripper tips or any obstacle presents between the gripper tip and the target

In the experiments of this study, the gripper tips were designed to be located symmetrically to the target during the telerobotic pinching operation. Therefore, subjects felt symmetrical proximity sensation on the two fingers (*i.e*., the thumb and the index finger) during the telerobotic pinch. However, in actual situations of telerobotic surgery, two gripper tips are often located asymmetrically to the target. Future studies can test how well the teleoperators do the voluntary adjustment using the asymmetric proximity sensation, to gain the symmetry between the two distances and to avoid unintended excessive force on one side. Further, any unexpected obstacle in between the gripper tip and the target object can cause another challenge to minimizing the initial contact force using the proximity sensation. Future studies also can test how well the teleoperators address the problem of unexpected obstacles, perhaps by the coordination between visual feedback and the proximity sensation.

## Conclusion

In this paper, we proposed to add the proximity sensation to the fingertip of the teleoperator, to improve the control accuracy of the telerobotic pinching operation. Particularly, the sensation was evoked electrically on the fingertip in action, with the pulsing frequency inversely proportional to the distance between the end effector and the target object. Since available sensory feedback (*i.e*., vision and proprioception) is not reliable for fine fingertip control during the approach phase of teleoperation, the additional sensory feedback on the site of actuation (*i.e*., fingertip in action) will be highly beneficial to enhance control accuracy. Such additional sensation can help teleoperators perform delicate telerobotic operations more accurately and safely, allowing them to locate effectors precisely to the objects and to significantly reduce the initial contact force and minimize the potential disturbance to subsequent manipulations. The proposed proximity sensation also occupies a minimal cognitive load, as suggested by the absence of additional time to approach and contact objects, allowing timely completion of telerobotic operations without unnecessary fatigue. Such a sensation at the periphery could be a significant addition to the telerobotic neurosurgery, which requires high control accuracy and safety. The application of the proximity sensation, however, is not limited to surgery but applicable to other telerobotic applications that need high control accuracy and delicacy at the approach, such as rescue missions or remote maintenance of structures in harsh environments such as space and underwater.

## Methods

### Human subject recruitment

The study was performed in accordance with relevant guidelines/regulations, according to the procedure described in the protocol approved by the Institutional Review Board of Texas A&M University (IRB2018-0893D). Eleven healthy human subjects participated in the study. The number of subjects was chosen based on previous studies on modulating fingertip cutaneous feedback to determine its effect on finger-grip force control^61,62^. Subjects consisted of three females and eight males. All subjects were over the age 18 with the average of 29.6. All subjects gave informed consent. All subjects were right-handed and used their right hand to perform experiments.

### Experiment procedure

#### Exp I: Customization of stimulation parameters for each subject and questionnaire to evaluate the perception

First, we customized stimulation amplitude and frequency for each subject based on verbal responses. Regarding amplitude, we set the minimum voltage level at where subjects reported any sensation (*i.e., perception threshold*), and set the maximum voltage level at where subjects reported any discomfort from the stimulation (*i.e., comfort threshold*), at the frequency of 100 *Hz*. To set the perception threshold, we started to apply the stimulation with very low intensity (5 *V*) and gradually increased it by 0.5 *V*, until subjects reported any sensation. We then kept increasing it by 0.5 *V* until subjects reported any discomfort, to set the comfort threshold. Regarding frequency, we searched the frequency range to evoke the pulsing sensation, while setting the amplitude at the mean value of the perception and comfort thresholds for each subject. The stimulation frequency was increased from 10 *Hz* by 5 *Hz* step until each subject reported a constant buzzing sensation and no more pulsing sensation. The frequency right before each subject started to feel the constant buzzing sensation was defined as *f*_*max*_. Therefore, subjects still felt the pulsing sensation at *f*_*max*_.

We then evaluated how each subject perceived the electrotactile feedback according to the frequency, via a questionnaire. Each subject was asked to fill out a questionnaire that consisted of five questions with a 5-point Likert scale. The first and second questions were to confirm if subjects felt a pulsing sensation at 10 *Hz* and (*f*_*max*_ − 5)*Hz*, respectively. The third question was to confirm if subjects still felt a constant buzzing sensation at (*f*_*max*_ + 5)*Hz*. The fourth question is to evaluate how differently subjects felt the frequency of pulsing between 10 *Hz* and (*f*_*max*_ − 5)*Hz*. The fifth question was to compare the intensity of sensation between 10 *Hz* and (*f*_*max*_ − 5)*Hz*.

#### Exp II: Test the efficacy of interventions (visual assistance and proximity sensation) on improving the control accuracy of telerobotic pinching

**Figure 7:**
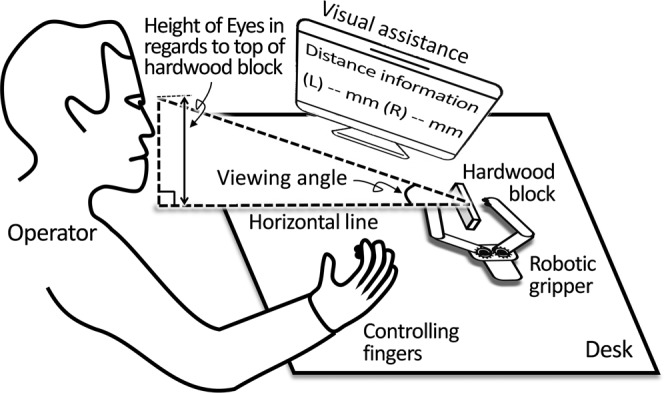
The experimental setup with a subject controlling the telerobotic gripper. It shows how visual assistance was given in the perspective of overall experimental setup. It also shows how the viewing angle is defined in regards to the center of operator’s eyes and the teleoperation site via top center of the hardwood block.

With the amplitude and frequency range of stimulation to evoke the pulsing sensation, customized for each subject, we tested the efficacy of the proximity sensation on minimizing the initial contact force and compared it with the efficacy of visual assistance. By receiving the data transmitted from two optical distance sensors attached onto the gripper tips, visual assistance displayed the distance between each gripper tip and the hardwood block on the monitor via numbers in the unit of millimeter, as described in Fig. 7. To compare the efficacy of two different interventions, the contact force was measured at each side of the hardwood block under three different conditions of sensory feedback: (1) vision only, (2) vision + visual assistance, and (3) vision + proximity sensation. *In the first condition*, subjects were provided with only visual feedback of the gripper tips*. In the second condition*, subjects were allowed to look at both the gripper tips and the monitor showing the numbers for distance. *In the third condition*, the proximity sensation was provided to the fingertips of the thumb and index finger with visual feedback on the gripper tips. All three tests were repeated with two different viewing angles: 30–60° and line-of-sight. Note that, the viewing angle is defined as the angle between the line connecting the top center of the hardwood block and the center of subjects’ eyes and the horizontal line on the desk, as seen in Fig. 7.

For all experimental conditions, the gripper tips were initially positioned symmetrical to the target, placing both tips at 15-mm distance from the target. Wearing the instrumented silicone finger cap and having bipolar gel electrodes on the fingertips, subjects were asked to apply minimal force at the contact. Additionally, subjects were asked to stop the pinching when they believed that contact was made and maintain the initial pinching force for 5 seconds. For each trial, the operator gave subjects a verbal sign to start pinching consistently when the operator clicked the start button for time ticking, to measure the time spent for the pinching. Each subject completed ten trials for each sensory condition with the order randomized to exclude any learning effect. This procedure of experiments was repeated for both viewing angles. Therefore, total number of trials for each subject was 60 (n = 30 for each viewing angle).

### Implementation of electronics and electrodes

*Silicone fingertip guards* were made to be worn on the index finger to measure the distance between the fingers, with the optical reflective sensor (HSDL9100-021, Broadcom) integrated onto the surface corresponding to the fingertip.

*Bipolar fingertip gel electrodes* were attached to the palmar digital nerve of each subject’s thumb and index finger, as seen in Fig. 3. The electrodes were custom made in the lab. Each electrode was composed of 1-*cm*2 area of a hydrogel, which adhered to a congruent sheet of conductive carbon fiber. This carbon fiber layer maintained robust electrical conduction to a multi-threaded 36-AWG wire via silver conductive epoxy.

*Robotic gripper* (ROT3U Robotic Clamp Claw, Aideepen) was selected as the slave arm, whose tips move similarly to human fingers for pinching operation. The servo motor (MG996R, Towerpro), used for gripper operation, was responsive enough to support the real-time telerobotic operation. A hard object was made from a hardwood block (10″×10″×1/4″) and firmly fixated onto the experiment table to keep the position and orientation of the target object between subjects. As shown in Fig. 3, two optical reflective sensors (HSDL9100-021, Broadcom) were installed onto each tip of the gripper, and covered by a transparent silicone elastomer (Sylgard 184, Dow Corning). The measurable range of the sensor was 0 to 60 *mm*, which covered the entire range of motion for the gripper tip.

*Microcontroller* (SAM3X8E, Microchip) received the distance information measured by optical distance sensors, and sent it to the operator’s thumb and index finger as an electrical stimulus delivering the proximity sensation. Microcontroller, along with the built-in stimulator, also generated a bi-phasic electrical stimulus with a pulse width of 1 *ms* for each polarity^63^.

*Two force sensors* (S15-4.5 N, SingleTact) were installed on each side of the hardwood block to detect the force applied by each tip of the gripper at initial contact. The full-scale force of the sensor was selected as 4.5 *N*, as a soft tapping force from the fingertip is reported as 0.5 to 1.47*N*^64^. The measurement resolution is 9*mN*, which could divide the reported soft-pinching force range into more than 100 steps.

### Data analysis

To evaluate the efficacy of each intervention, a two-tailed Welch’s t-test was performed at the 95% confidence level, as part of the datasets under comparison had unequal variances. To verify that the data satisfies the prerequisites for a t-test, we tested normality of data distribution using the Kolmogorov-Smirnov test of normality. All datasets satisfied the condition of p > 0.05 and normality could be assumed. We also applied Bonferroni correction considering nine comparisons were conducted with the same dataset, and then we used p < 0.0056 as the condition for statistical significance.

#### Comparison of initial contact force

**Figure 8:**
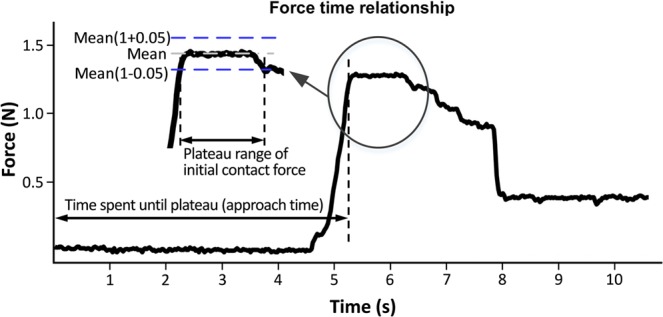
Graphical description of determining the initial contact force and the approach time, with one example of the measured contact force, averaged with two force outputs at both sides of the hardwood block.

We first identified the plat part of the force output after the initial change of the force output from the baseline (*i.e*., first plateau). Then, the range of the first plateau was identified by including the force output within ±5% of the mean value of the stable range of the force output in the middle of the plateau. The value after the first plateau was ignored to exclude the re-adjusted contact force after the contact was made. The force output during the plateau was averaged for each condition. For example, in the trial shown in Fig. 8, the plateau was identified between 5.25 *s* and 6.50 *s*, and the initial contact force was calculated as the mean value of the identified plateau range. The average and standard error of the initial contact force from eleven subjects were calculated for each condition, and the graphical representation along with the result of the statistical test are shown in Fig. 4a.

#### Comparison of approach time

We also identified the approach time required to reach the first plateau for each condition. For any single trial, the approach time was defined as the interval from the moment the operator gave the verbal signal of start to the subjects until the moment the waveform reached the first plateau (see Fig. 8). The average and standard error of the approach time from the eleven subjects were calculated for each condition, and the graphical representation along with the result of the statistical test are shown in Fig. 4b.

## Data Availability

The datasets generated during and/or analysed during the current study are available from the corresponding author on reasonable request.
